# Genome-wide identification, characterization and expression analysis of the *bZIP * transcription factors in garlic (*Allium sativum* L.)

**DOI:** 10.3389/fpls.2024.1391248

**Published:** 2024-08-01

**Authors:** Shutao He, Sen Xu, Zhengjie He, Xiaomeng Hao

**Affiliations:** ^1^ Institute of Neurobiology, Jining Medical University, Jining, China; ^2^ Institute of Biotechnology and Health, Beijing Academy of Science and Technology, Beijing, China; ^3^ Rehabilitation Department, Traditional Chinese Medicine Hospital of Yanzhou District of Jining City, Jining, China

**Keywords:** genome-wide, garlic, bZIP, abiotic stress, expression profiles

## Abstract

**Introduction:**

The *bZIP* genes (*bZIPs*) are essential in numerous biological processes, including development and stress responses. Despite extensive research on *bZIPs* in many plants, a comprehensive genome-wide analysis of *bZIPs* in garlic has yet to be undertaken.

**Methods:**

In this study, we identified and classified 64 *AsbZIP* genes (*AsbZIPs*) into 10 subfamilies. A systematic analysis of the evolutionary characteristics of these *AsbZIPs*, including chromosome location, gene structure, conserved motifs, and gene duplication, was conducted. Furthermore, we also examined the nucleotide diversity, cis-acting elements, and expression profiles of *AsbZIPs* in various tissues and under different abiotic stresses and hormone treatments.

**Results and Discussion:**

Our findings revealed that gene replication plays a crucial role in the expansion of AsbZIPs, with a minor genetic bottleneck observed during domestication. Moreover, the identification of cis-acting elements suggested potential associations of *AsbZIPs* with garlic development, hormone, and stress responses. Several *AsbZIPs* exhibited tissue-preferential and stress/hormone-responsive expression patterns. Additionally, *Asa7G01972* and *Asa7G01379* were notably differentially expressed under various stresses and hormone treatments. Subsequent yeast two-hybridization and yeast induction experiments validated their interactions with *Asa1G01577*, a homologue of ABI5, reinforcing their importance in hormone and abiotic stress responses. This study unveiled the characteristics of the *AsbZIP* superfamily and lays a solid foundation for further functional analysis of *AsbZIP* in garlic.

## Introduction

Transcription factors (TFs) play pivotal roles in regulating gene expression by recognizing and binding to promoters, which is essential for plant development and stress responses (Singh et al., 2002; Li et al., 2013). The basic leucine zipper (bZIP) family is one of the TF families and is widely present in eukaryotes (Rodriguez-Uribe and O’Connell, 2006; Nijhawan et al., 2008). The bZIP family is characterized by a conserved bZIP domain, usually comprising 60 to 80 amino acids, which contains a basic region and a leucine zipper region (Nijhawan et al., 2008). The basic region, positioned at the N-terminal end of the region, contains a conserved N-x7-R/K motif that is related to nuclear localization and binds to target DNA (Suckow et al., 1994; Correa et al., 2008). The leucine zipper region exhibits a relatively lower level of conservation, consisting of a repetitive sequence comprising leucine or other hydrophobic amino acids (Ile, Val, Phe, or Met). Specifically, 9 of these amino acids are positioned at the C-terminus, forming an amphipathic helix (Jakoby et al., 2002; Wei et al., 2012; Liu and Chu, 2015; Hu et al., 2016b).

The *bZIP* gene family has been comprehensively characterized and investigated at the genomic level across various plant species, including *Arabidopsis thaliana* (Jakoby et al., 2002), *Oryza sativa* (Nijhawan et al., 2008), *Glycine max* (Liao et al., 2008b), *Sorghum bicolor* (Wang et al., 2011), *Zea mays* (Wei et al., 2012), *Vitis vinifera* (Liu et al., 2014b), *Cucumis sativus* (Baloglu et al., 2014), *Maninot esculenta* (Hu et al., 2016b), *Malus domestica* (Zhao et al., 2016), *Brassica napus* (Zhou et al., 2017), *Fragaria ananassa* (Wang et al., 2017), *Daucus carota* (Que et al., 2015) and *Hordeum vulgare* (Pourabed et al., 2015). Previous studies have demonstrated the important role of *bZIPs* in diverse crucial biological processes, such as organ and tissue differentiation (Gangappa et al., 2013; Lozano-Sotomayor et al., 2016; Zhang et al., 2016; Tan et al., 2020), seed storage (Bombarely et al., 2012; Edwards et al., 2017), metabolic activity (Baena-Gonzalez et al., 2007), photomorphogenesis and light signal regulation (Joo et al., 2014; Babitha et al., 2015), salt and drought tolerance (Ying et al., 2012; Liu et al., 2014a), and hormone and sugar signaling processes (Fan et al., 2019; Liu et al., 2020). Specifically, *HY5*, a key transcription factor in light signal transduction, encodes a bZIP protein that regulates root and hypocotyl development in *Arabidopsis thaliana* (Oyama et al., 1997). *AtZIP1* is involved in sugar signaling and influences seed growth and development (Wigge et al., 2005). Additionally, AtbZIP53 can form heterodimers with AtbZIP1, 10 or 25 to promote the transcriptional activation of seed maturation genes (Alonso et al., 2009). *AtZIP17* and *AtZIP24* are crucial in the salt stress response (Liu et al., 2008; Yang et al., 2009). In rice, *OsbZIP46* is strongly upregulated under drought, heat, and abscisic acid (ABA) stresses (Tang et al., 2012). *OsbZIP72* is induced by drought and ABA treatments (Lu et al., 2009). In addition, *SlbZIP33* is involved in stress-induced response and plays a vital role in fruit ripening (Orellana et al., 2010; Bastias et al., 2014). *GmbZIP15* increases the sensitivity of soybean to salt and drought stresses by negatively regulating the gene expression levels of *GmWRKY12* and *GmABF1* (Zhang et al., 2020a). *IbbZIP1* strongly responds to ABA and is related to salt and drought tolerances in sweet potato (Kang et al., 2019). *TabZIP6* is involved in cold tolerance by forming a dimer with two other bZIP proteins belonging to the S subfamily (Cai et al., 2018).

Garlic, originating from Central Asia, the Mediterranean and the Caucasus, has been cultivated for over 5,000 years. Like onion, which is the largest crop in the *Allium* genus, garlic is not only an economically important vegetable and spice, but is also widely applied in the pharmaceutical and nutritional industries (ETOH et al., 2001; Martin and Ernst, 2003; Kamenetsky et al., 2015). At present, due to global warming and the shrinking availability of cultivated land, enhancing the quality of key traits is particularly imperative for garlic breeding. *bZIP* genes play vital roles in numerous physiological processes; thus, comprehensive identification and analysis of *bZIP* gene family members are essential. However, a notable research gap exists in the investigation of *bZIP* genes in garlic. The successful assembly of the garlic chromosome genome provides a solid foundation for these endeavors (Sun et al., 2020).

In this work, 64 *AsbZIP* genes (*AsbZIPs*) were identified and separated into 10 groups on the basis of phylogenetic relationships; these groups were compared with those of *Arabidopsis thaliana* and *Oryza sativa*, which are representative species of dicotyledonous and monocotyledonous plants, respectively. Further comprehensive analyses of *AsbZIPs*, including gene structure, motif analysis, chromosome distribution, evolutionary characteristics and *cis*−acting elements, were conducted. Additionally, we investigated the variation atlas of single-nucleotide polymorphisms (SNPs) in *AsbZIPs*. Finally, we explored the expression profiles of *AsbZIPs* in different garlic tissues and under multiple stresses via quantitative real-time polymerase chain reaction (qRT-PCR). Our study provides a solid foundation for further functional investigations of *AsbZIPs*.

## Materials and methods

### Identification of *bZIP* genes in the garlic genome

Whole garlic genome data were downloaded from https://doi.org/10.6084/m9.Figshare.12570947.v1, and those of *Arabidopsis thaliana* and *Oryza sativa* were retrieved from the TAIR database (https://www.Arabidopsis.org/) and the RGAP database (https://rice.plantbiology.msu.edu/), respectively. The bZIP domains (PF00170 and PF07716) were retrieved from the PFAM database (http://pfam.xfam.org) (El-Gebali et al., 2019) and used to perform an HMM (hidden Markov model) search via the HMMER 3.0 program. NCBI-CDD (https://www.ncbi.nlm.nih.gov/cdd/) and SMART (http://smart.embl.de/) were used to further confirm the bZIP domain of potential *bZIP* gene family members. The ExPASy proteomics server (https://web.expasy.org/computepi/) was used to calculate the molecular weight (MW), isoelectric point (pI), instability index (I.I.), aliphatic index (A.I.), total number of negatively charged residues (Asp + Glu, n.c.r, %), total number of positively charged residues (Arg + Lys, p.c.r, %), and grand average hydropathicity (GRAVY) of the bZIP proteins in garlic. The subcellular localizations of the bZIP proteins were assessed via the Cell-PLoc 2.0 web server (http://www.csbio.sjtu.edu.cn/bioinf/plant-multi/).

### Phylogenetic analysis

The bZIP protein sequences from *Allium sativum*, *Arabidopsis thaliana*, and *Oryza sativa* were aligned via MUSCLE with default parameters. A phylogenetic tree for these proteins was established via the neighbor-joining (NJ) method in MEGA 7.0 software (Kumar et al., 2018), with 1000 iterations. The iTOL online software tool (https://itol.embl.de/) was used to output visual images.

### Gene structure and conserved motif analysis

The Gene Structure Display Server (GSDS) (http://gsds.gao-lab.org/) (Hu et al., 2015) was employed to identify the exon and intron structures of all *AsbZIPs*. The MEME program (https://meme-suite.org/meme/) (Bailey et al., 2009) was used to investigate conserved motifs. The minimum and maximum lengths of the conserved motifs were set to 6 and 50, respectively, with a maximum of 10 conserved motifs.

### Chromosomal localization and gene duplication analysis of *AsbZIPs*

The chromosomal positions of the *AsbZIP* genes were visualized via MapChart software (Stajich et al., 2002) according to the garlic genome annotation (https://doi.org/10.6084/m9.Figshare.12570947.v1). Using the Multiple Collinear Scan Toolkit (MCScanX), we investigated the gene duplication events of the *AsbZIP* genes (Wang et al., 2012). A tandem duplication event is a chromosome region with two or more adjacent genes within a 200 kb range, and duplicated pairs positioned on different chromosomes are defined as segmental duplication events (Cannon et al., 2004). Syntenic analysis of *bZIP* genes between garlic and four plant species (*Arabidopsis thaliana*, *Oryza sativa*, *Zea mays*, and *Theobroma cacao*) was conducted via Dual Synteny Plotter software (https://github.com/CJChen/TBtools) (Chen et al., 2020). The calculation of synonymous (Ks) and nonsynonymous (Ka) substitution rates and Ka/Ks ratios of each duplicated gene pair were carried out based on the coding sequences (CDS) alignments of *bZIP* genes via Ka/Ks calculator 2.0 software, and a Ka/Ks ratio > 1 was interpreted as positive selection, < 1 as purified selection, and = 1 as neutral evolution (Nei and Gojobori, 1986; Wang et al., 2010).

### Analysis of *cis*-acting elements

*Cis*-acting elements within the 2000 bp region upstream of the transcriptional start site of each *AsbZIP* gene were identified via the PlantCARE database (http://bioinformatics.psb.ugent.be/webtools/plantcare/html) (Chow et al., 2019).

### Nucleotide variation and population structure

The resequencing data of 233 garlic samples were retrieved from the Genome Variation Map (project accession: PRJCA006629). **Supplementary Table 1** presents the geographic distribution and detailed material information. SnpEff v4.3 was utilized for the annotation of SNPs (Cingolani et al., 2012). Additionally, the population structure was analyzed via ADMIXTURE v1.3.0, with K values ranging from 2 to 5. The phylogenetic tree was established by Treebest v1.9.2, and the Smartpca within EIGENSOFT v4.2 was used for the analysis of principal component analysis (PCA). Nucleotide diversity (π) and Wright’s F statistic (Fst) were calculated using VCFtools v0.1.16.

### Plant materials and various stresses and hormone treatments

Garlic cloves (cv. Ershuizao) were planted in pots and cultivated in a chamber (16 h/8 h of light/dark, 30°/22° day/night). The treatment experiments were performed as described previously (Zhang et al., 2022b; Munim Twaij et al., 2023; Yao et al., 2023; Shi et al., 2024) with some modifications. The salt, cold and heat stress experiments were simulated with a 200 mM NaCl solution, at 4° and 30°, respectively. Five hormone treatments, including ABA (1 mg/L), GA3 (gibberellic acid, 200mg/L), MeJA (methyl jasmonic acid, 100 µM), IAA (indoleacetic acid, 100 µM), and SA (salicylic acid, 100 µM)), were conducted. Root and leaf samples were collected at 0, 6, 12, 24 and 48 hours after each treatment and promptly frozen in liquid nitrogen. For the drought treatment, irrigation was ceased for the seedlings in the treatment group, and those in the control group were irrigated normally. Roots and leaves were harvested at 0, 7 and 14 days after treatment. Additionally, freshly harvested garlic cloves were stored at 4°C and collected at 0, 10, 15, and 40 days after treatment. To investigate the tissue-specific expression profiles of the *AsbZIPs*, leaves, stems, pseudostems, roots, buds, bulbs and flowers were harvested at 192 days after planting. All samples were promptly frozen in liquid nitrogen and then preserved at -80°C for subsequent mRNA extraction.

### RNA isolation and qRT-PCR analysis

RNA extraction kits (Vazyme, Nanjing, China) were used to extract total RNA according to the manufacturer’s instructions. Subsequently, reverse transcription of two micrograms of RNA was conducted via HiScript III RT SuperMix for qPCR (Vazyme, Nanjing, China). The qRT-PCR assay was conducted as previously described (Xu et al., 2013). **Supplementary Table 2** shows the primers designed with Primer Express software (v3.0). The *tubulin* gene was used as the internal control gene, and the expression level of each gene was determined via the 2^−ΔΔCt^ method (Livak and Schmittgen, 2001).

### Gene regulatory network analysis

The co-expression network was constructed via 185 RNA-Seq datasets retrieved from the Gene Expression Omnibus (GEO) database (accession codes GSE211495, GSE186042, and GSE145455) and the Sequence Read Archive (SRA) database (accession codes PRJNA682570, PRJNA472416, and PRJNA683607). The high-quality reads were filtered via the NGS QC Toolkit (v2.3). TopHat (v2.0.0) was used to align these filtered reads to the garlic genome with default parameters. Then, the FPKM values and read counts of each garlic gene were calculated via Cufflinks (v2.0.2). To identify genes co-expressed with *AsbZIP* genes, weighted gene co-expression network analysis (WGCNA) was used to construct a co-expression network. For further investigation, genes within the top 5% highest weighted values associated with *AsbZIP* genes were selected.

Furthermore, to determine directional interactions in the transcriptional regulatory network related to *AsbZIP* genes, 2-kb upstream sequences of co-expressed genes of each *AsbZIP* gene were analyzed, and FIMO (Grant et al., 2011) was utilized to screen genes whose promoters included a significantly enriched motif of corresponding *AsbZIP* gene according to the high-quality TF binding motifs retrieved from the PlantTFDB database. A motif was regarded as present in a promoter if it had at least one match at a *P* value ≤ 10^-4^. The clusterProfiler package in R was used to perform GO enrichment analysis of the filtered co-expressed genes of each *AsbZIP* gene.

### Prediction of the AsbZIP protein interaction network

STRING (https://cn.string-db.org/) was used to construct an interaction network for AsbZIP proteins according to their orthologous proteins in *Arabidopsis thaliana*.

### Heterologous expression in yeast and yeast two-hybrid

To investigate the functions of *Asa7G01972* and *Asa7G01379* in salt stress, a specific primer pair for each gene (**Supplementary Table** **2**) was used to amplify the DNA fragment, which was cloned in frame into the pYES2 recombinant vector. The recombinant and empty vector plasmids were separately transformed into yeast cells (BY4741) following the manufacturer’s protocol for the Yeastmaker™ Yeast Transformation System 2 (Clontech Laboratories, Inc., Palo Alto, CA, USA). Yeast transformants were cultivated in SD/-Ura liquid medium at 30° until the OD_600_ reached 0.5. The preculture was transferred to SG (-Ura, 2% galactose) and diluted to an OD_600_ of 0.4, followed by incubation with shaking for an additional 24 hours at 30°C to induce gene expression. Subsequently, the yeast cells were harvested for subsequent stress treatments (Ibrahim et al., 2001).

The Y2H experiment was conducted according to the manufacturer’s instructions (Clontech Laboratories, Inc., Palo Alto, CA, USA). The CDS of *Asa7G01972* or *Asa7G01379* was inserted into the pGBKT7 vector to form the bait construct, whereas that of *AsaABI5* (geneID: *Asa1G01577*) was cloned and inserted into the pGADT7 vector to generate the prey construct. The primers used for the Y2H assay are presented in **Supplementary Table 2**. These plasmids were then transformed into yeast cells (strain AH109), and the resulting yeast transformants were cultured on SD medium supplemented with 3-aminotriazole and X-α-gal but lacking tryptophan, leucine, histidine, and adenine at 30°C for 2−3 days.

### Statistical analysis

The samples were harvested from three independent plants. Data from at least three replicates are shown as the means ± SDs. The statistical analysis, including Student’s *t* test, was conducted via SPSS software (version 17, SPSS Inc., Chicago, IL, USA). A significance criterion of *P* < 0.05 indicated statistical significance.

## Results

### Identification and characterization of *bZIP* genes in garlic

A total of 64 *AsbZIP* genes were identified via hmm search and confirmed via the NCBI-CDD and SMART databases (**Supplementary Table** **3**). Apart from 3 genes (*Asa0G04894*, *Asa0G01277*, and *Asa0G02642*) located on unassigned scaffoldings, the remaining 61 genes were randomly distributed across 8 chromosomes. Specifically, chromosome 4 had the greatest number of genes, with 12, whereas chromosome 3 had the lowest number of genes, with only 4.

Gene characteristics were further analyzed. The CDS lengths of the *AsbZIP* genes ranged from 240 bp (*Asa2G04074*) to 1758 bp (*Asa8G00330*), with predicted molecular weights (MWs) ranging from 9.24256 to 64.00282 kDa. Asa7G01972 and Asa7G01871 presented the extremes in terms of isoelectric point (pI), with values of 4.89 and 11.24, respectively, indicating the lowest and highest pIs within the set. All the *AsbZIP* proteins had consistently negative GRAVY values, indicating their hydrophilic nature, which was coincident with their presumed roles as transcription factors. Apart from 8 genes (*Asa3G03771*, *Asa4G00594*, *Asa4G00875*, *Asa4G04645*, *Asa5G05838*, *Asa7G02467*, *Asa7G05774*, and *Asa8G00330*), the majority of proteins exhibited instability. Furthermore, all the AsbZIP proteins were localized in the nucleus.

### Phylogenetic analysis and classification of *AsbZIPs*

**Figure 1:**
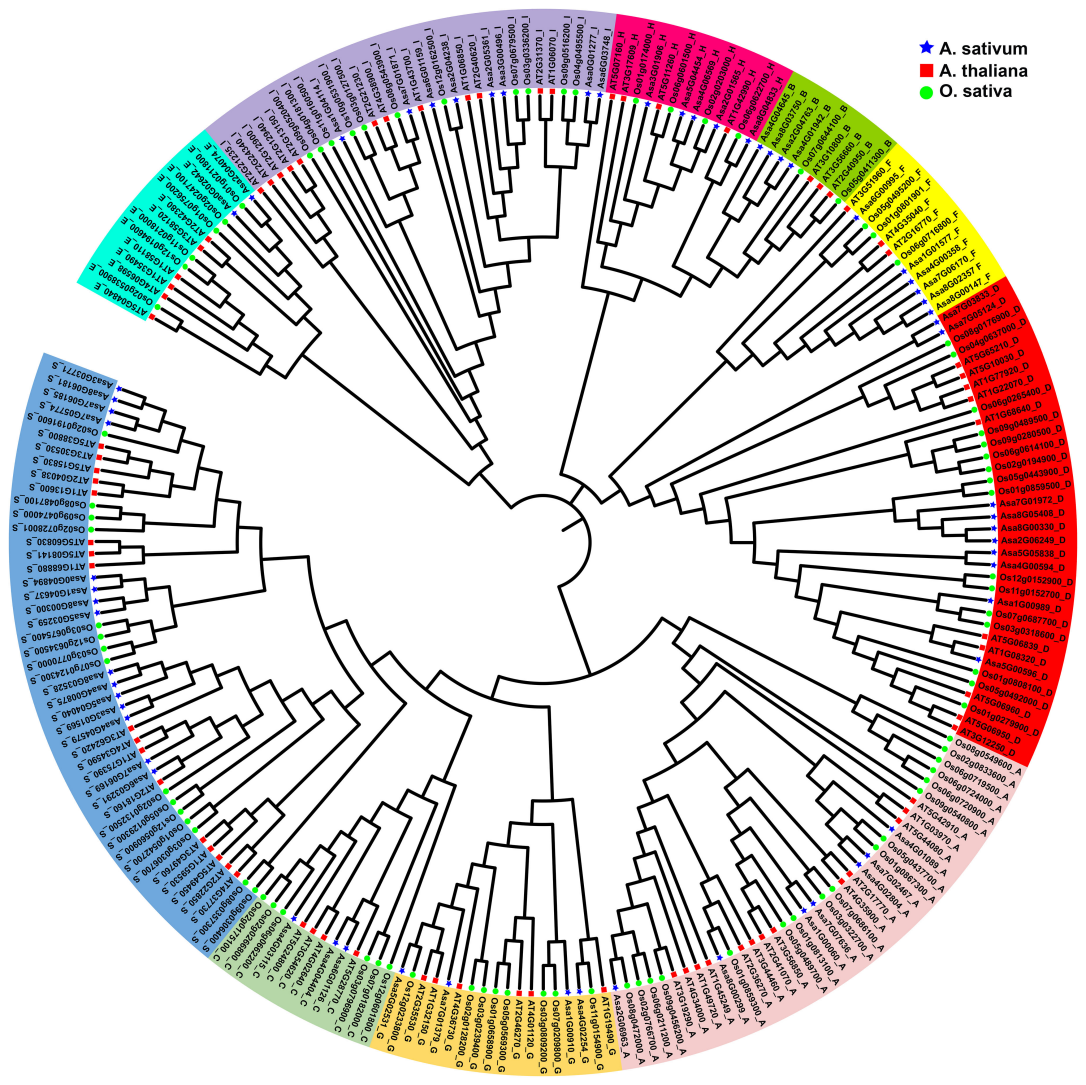
Phylogenetic relationships of the identified bZIP proteins in *Allium sativum*, *Arabidopsis thaliana*, and *Oryza sativa*. The colored regions represent *bZIP* genes of different subfamilies A to I, S. The blue stars indicate the AsbZIP proteins, the red squares represent the AtbZIP proteins, the green circles represent the OsbZIP proteins.

bZIP protein sequences from garlic (64), *Arabidopsis thaliana* (78), and *Oryza sativa* (88) were used to construct a phylogenetic tree via the NJ method, delineating their evolutionary relationships (**Figure 1**). A total of 230 bZIPs from these species were divided into 10 subfamilies, labelled as Groups A, B, C, D, E, F, G, H, I, and S, on the basis of their classification in *Arabidopsis thaliana* (Jakoby et al., 2002). The subfamilies varied in size, with the largest encompassing 47 members (S group) and the smallest containing 9 members (B group). Notably, all the species contributed members to each identified subfamily.

### Gene structure and conserved motif analysis

**Figure 2:**
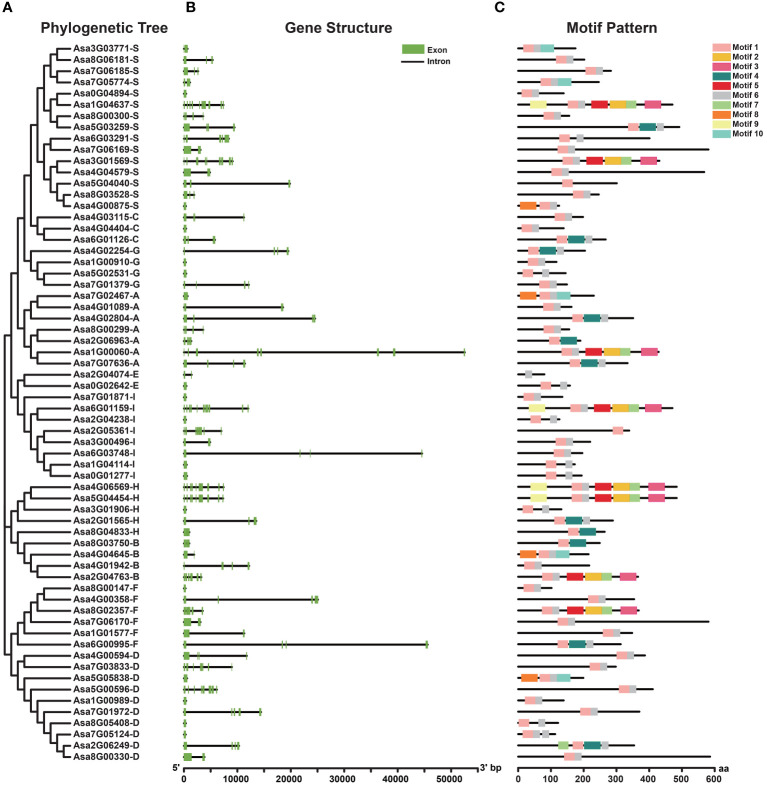
Phylogenetic relationships, gene structures and conserved protein motifs of the *AsbZIP* superfamily in garlic. **(A)** Phylogenetic relationships of 64 AsbZIP proteins. **(B)** Exon-intron structure of *AsbZIP* genes. Green boxes indicate exons, and black lines indicate introns. **(C)** The motif profile of AsbZIP proteins. These motifs are presented in differently colored boxes numbered 1-10. The sequence information of each motif is provided in **Supplementary Table** **4**. The protein length can be estimated using the scale at the bottom.

The structure of exons and introns provides pivotal evidence for discerning phylogenetic relationships within gene families (Li et al., 2006). The number and distribution of exons and introns of *AsbZIPs* were investigated (**Figures 2A, B**). The results revealed that 17 *AsbZIPs*, constituting 27% of the total, lacked introns, and Groups D and I had the most abundant intron-lacking *AsbZIP* genes, with four. Among *AsbZIPs* with introns, the number of introns ranged from 1 to 11. The greatest number of introns among the *AsbZIP* genes was 11, and these genes belonged to Groups A, D, H, S and I. The *AsbZIPs* in Groups C and G contained either 0 or 3 introns, whereas those in Group E had either 0 or 1 introns. Generally, *AsbZIP* genes within the same subfamily presented similar gene structures.

Additionally, to gain insight into the divergence and characterization of AsbZIP proteins, 10 conserved motifs were identified (**Figures 2A, C**). These motifs varied in length, ranging from 21 (motif 6) to 50 (motifs 2, 3, 4, 5, 8 and 9) (**Supplementary Table** **4**). The majority (89.06%) of the AsbZIP proteins presented the prevalent presence of motifs 1 and 6. Motif 8 was present in Groups A, B, D and S; motif 9 was found in Groups H, I and S; and motif 10 occurred in Groups A, B and S. Numerous motifs were found in particular groups, suggesting potential associations with distinct biological functions.

### Chromosomal distribution, gene duplication events and synteny analysis of *bZIPs* in garlic

**Figure 3:**
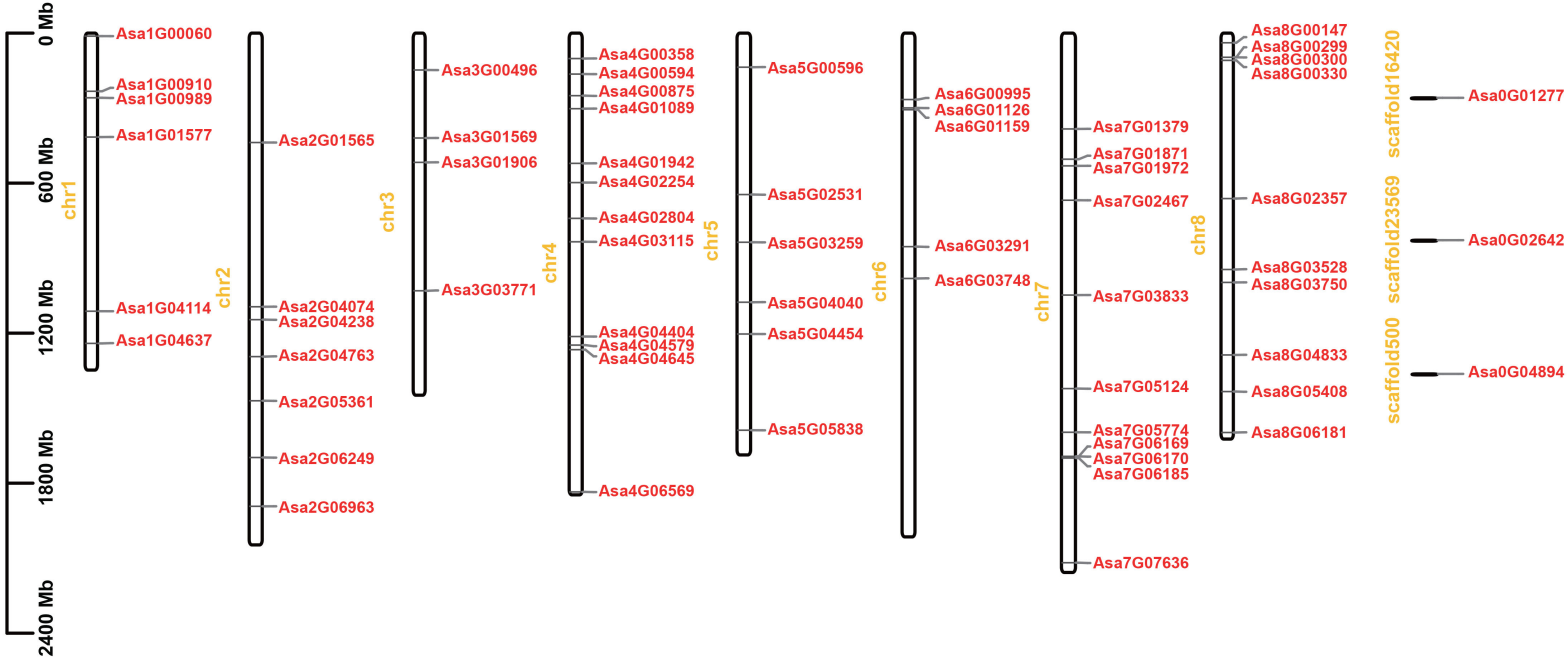
Chromosomal distribution of *bZIP* genes in garlic. The chromosome number is marked on the left of each chromosome in yellow. Chromosome lengths and gene positions can be inferred from the scale on the left.

A total of 61 *AsbZIP* genes were unevenly distributed on 8 chromosomes, and three *AsbZIP* genes (*Asa0G01277, Asa0G02642*, and *Asa0G04894*) were present on the scaffolds (**Figure 3**). Chromosome 4 had the most genes, with 12 genes, followed by chromosome 7 (11 genes), chromosome 8 (10 genes) and chromosome 2 (7 genes). Chromosome 1 and chromosome 5 each featured an identical number of 6 *AsbZIP* genes. Chromosome 3 had the lowest number of *AsbZIP* TFs (4 genes).

**Figure 4:**
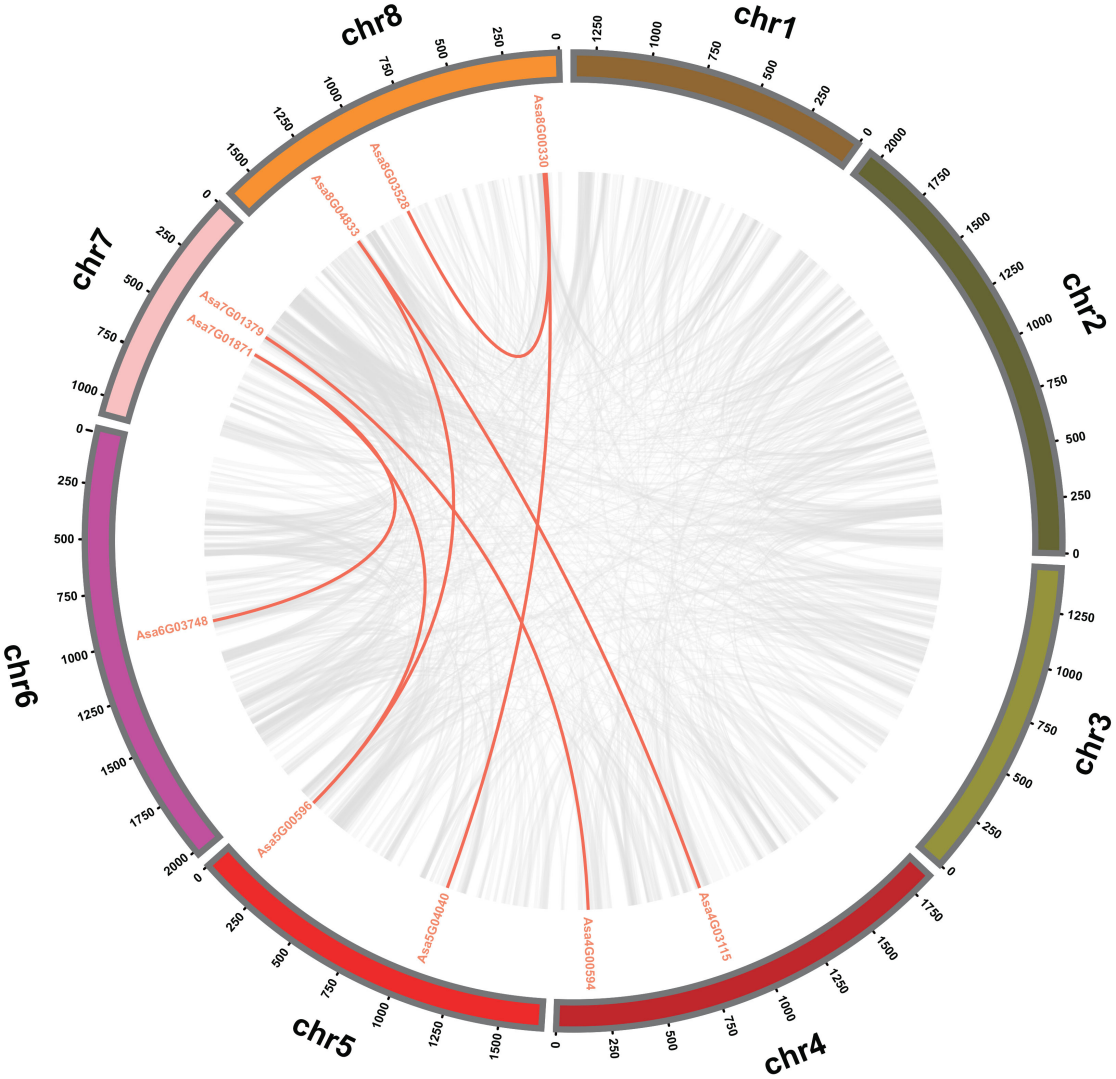
Interchromosomal relationships of the *bZIP* genes in garlic. Different-colored circles represent distinct chromosomes, with grey lines within the circles representing all syntenic blocks in the garlic genome and red lines within the circles indicating collinear blocks among *AsbZIP* genes.

To explore the evolutionary patterns of the *AsbZIP* genes, tandem and segmental duplication events were analyzed. Two tandem duplication regions, *Asa7G06170* and *Asa7G06169*, *Asa8G00299* and *Asa8G00300*, are located on chromosomes 7 and 8, respectively (**Figure 3**; **Supplementary Table** **5**). Furthermore, seven pairs of segmental duplicated genes (*Asa4G00594* and *Asa7G01379*, *Asa5G00596* and *Asa7G01871*, *Asa6G03748* and *Asa7G01871*, *Asa4G03115* and *Asa8G04833*, *Asa5G00596* and *Asa8G04833*, *Asa5G04040* and *Asa8G00330*, *Asa8G03528* and *Asa8G00330*) were identified; these pairs were associated with chromosomes 4 and 7, chromosomes 5 and 7, chromosomes 6 and 7, chromosomes 4 and 8, chromosomes 5 and 8, chromosomes 5 and 8, and chromosome 8, respectively (**Figure 4**; **Supplementary Table** **5**). These observations strongly suggest that tandem and segmental duplication played a significant role in the expansion of *AsbZIP* genes.

To delve into the evolutionary restrictions of *AsbZIP* genes, the Ka *vs*. Ks substitution ratios were analyzed (**Supplementary Table** **5**). The Ka/Ks ratios between *Asa7G06170* and *Asa7G06169*, *Asa8G00299* and *Asa8G00300*, *Asa8G00330* and *Asa8G03528*, *Asa8G00330* and *Asa5G04040*, *Asa7G01871* and *Asa6G03748*, and *Asa7G01871* and *Asa5G00596* were less than 1, implying purifying selection, whereas those between *Asa8G04833* and *Asa5G00596*, *Asa8G04833* and *Asa4G03115*, and *Asa7G01379* and *Asa4G00594* were greater than 1, suggesting positive selection.

### Evolutionary analysis of *AsbZIP* genes and several other species

**Figure 5:**
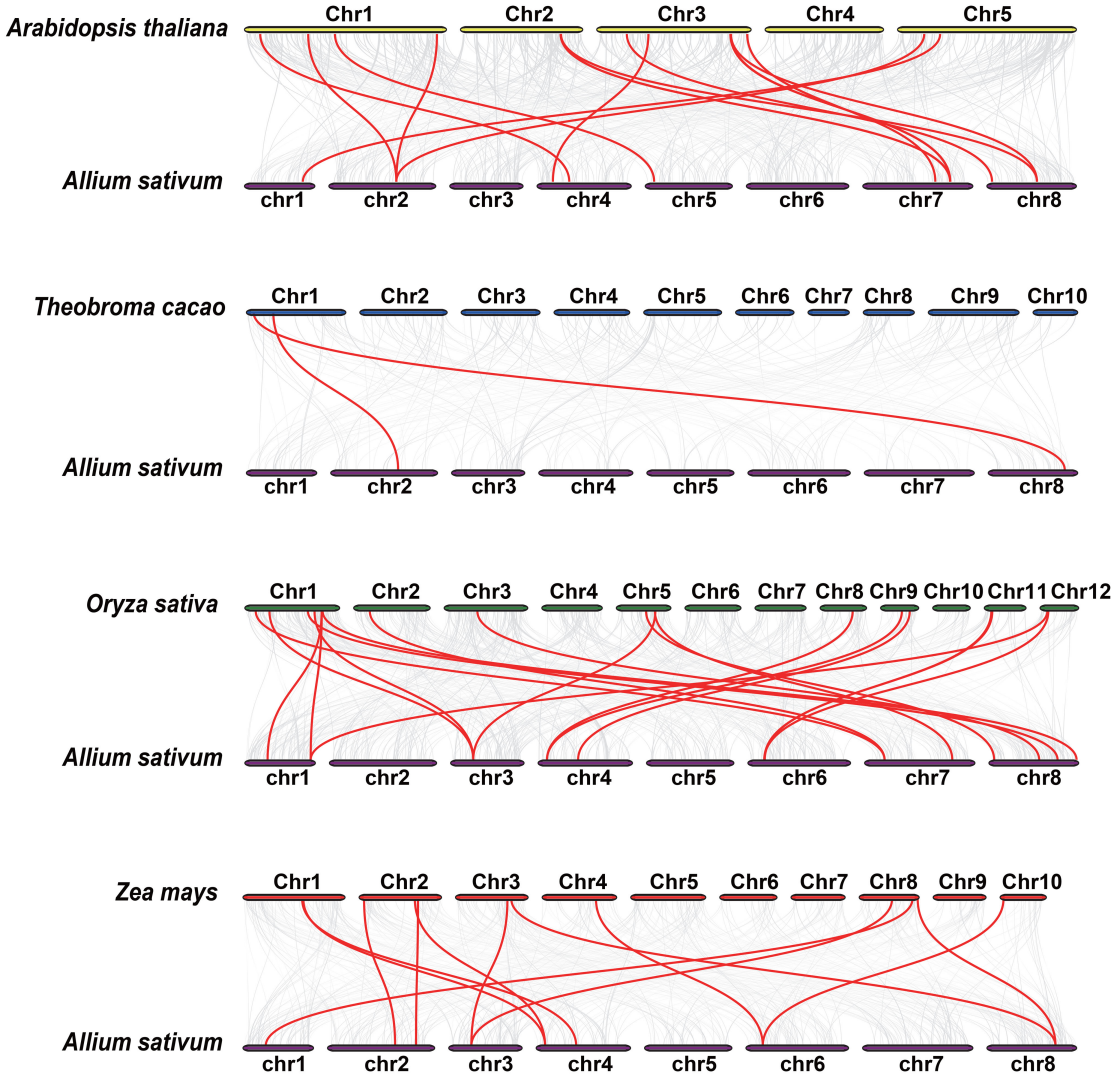
Syntenic analysis of *bZIP* genes between garlic and four representative plant species. Gray lines in the background represent collinear blocks between garlic and other plant genomes, whereas red lines highlight syntenic *bZIP* gene pairs.

To further elucidate the phylogenetic mechanisms of the *AsbZIP* family, we established syntenic maps of garlic compared with those of four other representative species: two monocotyledonous plants (*Oryza sativa* and *Zea mays*) and two dicotyledonous plants (*Arabidopsis thaliana* and *Theobroma cacao*) (**Figure 5**). Twenty-two *bZIP* genes in garlic exhibited collinearity associations with those in *Oryza sativa* (19 pairs), *Zea mays* (12 pairs), *Arabidopsis thaliana* (15 pairs), and *Theobroma cacao* (2 pairs). Additionally, 19 gene pairs consisting of only 6 *AsbZIPs* were detected between garlic and *Oryza sativa*, and *Zea mays*, indicating that these orthologous pairs appeared after the divergence between monocotyledonous and dicotyledonous plants.

### Nucleotide variation and population structure of *AsbZIP* genes

Publicly available garlic resequencing data were used to explore *AsbZIP*-related SNPs, revealing the intricate sequence diversity of *AsbZIP* genes. The dedicated SNP calling pipeline successfully generated 2941 SNPs, each with high confidence (**Supplementary Table** **6**). A predominant portion of *AsbZIP*-associated SNPs were enriched in intergenic regions, whereas others occurred within genic regions, including 4 missense, 40 intron, 1 synonymous, 43 downstream, and 70 upstream variants. The overall transition/transversion (Ts/Tv) ratio was 1.938, with G/A (21.73%) and C/T (19.25%) as the predominant allelic substitution patterns, indicating a higher frequency of mutations involving purine-to-purine or pyrimidine-to-pyrimidine transitions than mutations that switch pyrimidines to purines or purines to pyrimidines.

**Figure 6:**
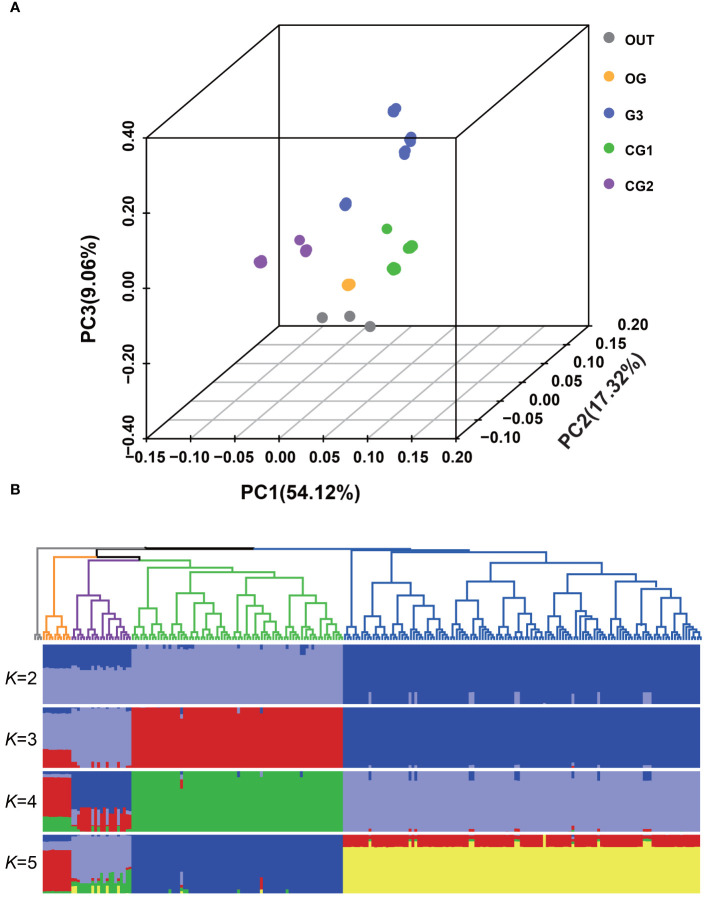
Population structure of wild and local garlic based on *AsbZIP*-related SNPs. **(A)** Plots of principal component analysis for the first (PC1), second (PC2), and third (PC3) components. Dot color indicates population and location. **(B)** Phylogenetic tree and population structure with *K* ranging from 2 to 5. The rooted tree was constructed using neighbor-joining method. The orders and positions of sample accessions on the x-axis are consistent with those in the neighbor-joining tree.

PCA was performed using *AsbZIP*-related SNPs to explore the interrelationship among the origin group and three distinct garlic cultivars (**Figure 6A**). The first eigenvector, which explained 54.12% of the total genetic variance, indicated divergence within these populations. The second and third eigenvectors, contributing 17.32% and 9.06% to genetic variation, respectively, facilitated the differentiation among these groups. The phylogenetic tree displayed consistent population affinities (**Figure 6B**). In accordance with the phylogenetic tree, ADMIXTURE analysis also validated the consistent group relatedness. At *K*=4, a clear demarcation according to geographical origin was observed. The existence of a genetic mixture between wild and landrace garlic indicated a possible domestication of cultivated garlic and the ongoing gene flow between wild and landrace garlic.

To assess the occurrence of genetic bottlenecks in *AsbZIPs* in garlic during acclimation, the population-based nucleotide diversity of *AsbZIP* genes was calculated. *AsbZIP* genes presented a slight decrease of only 0.00003% in nucleotide diversity from wild garlic to local garlic (**Supplementary Figure 1**), suggesting a weak genetic bottleneck during domestication. Additionally, we measured the degree of population differentiation via Wright’s F statistic. The calculated Fst index of 0.2235 between wild and landrace garlic within the *AsbZIPs* indicated that this gene family experienced relatively mild selective pressures during the domestication of garlic.

### Analysis of *Cis*−acting elements in *AsbZIP* promoters

**Figure 7:**
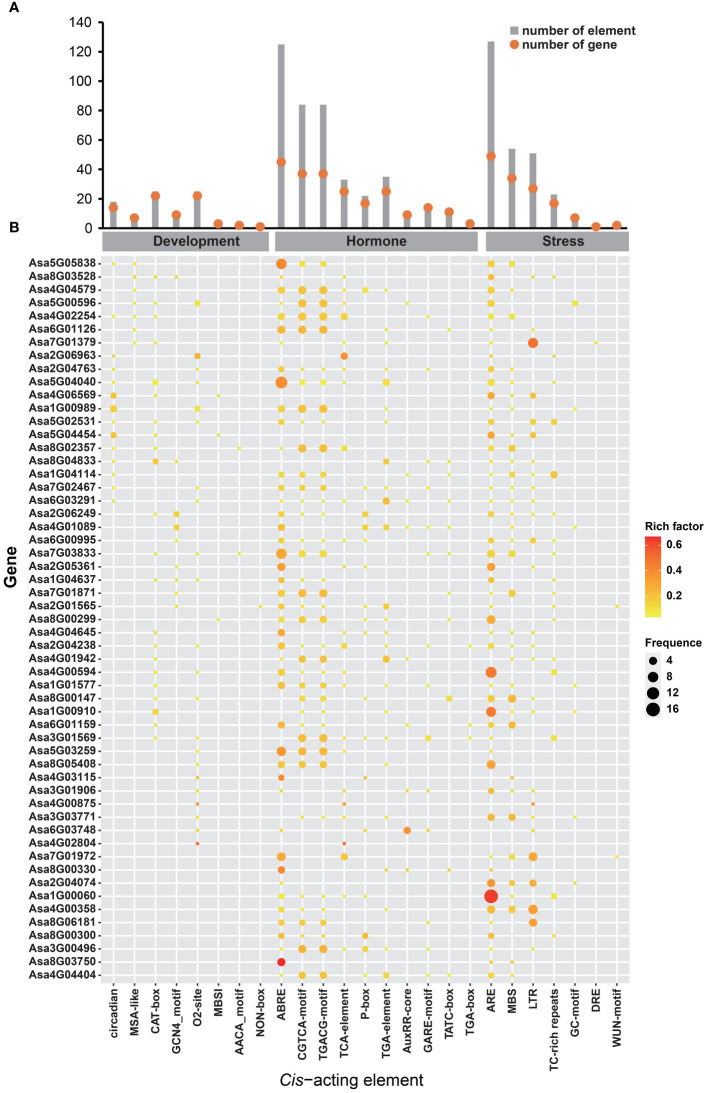
*Cis*-acting elements in the promoter of *AsbZIP* genes. **(A)** Number of each *cis*-acting element in the promoter regions of the *AsbZIP* genes. Red dots indicate the number of genes containing the corresponding *cis*-acting element, while gray boxes represent the total number of *cis*-acting elements. **(B)** Frequency of each *cis*-acting element in the promoters of *AsbZIP* genes. *Cis*-acting elements were categorized into development-related, hormone-related, and stress-related classes.

Regulatory mechanisms potentially regulating *AsbZIP* genes were investigated by analyzing the *cis*-elements present in their promoters. The results revealed that these *cis*-elements could be classified into three groups: development, hormone and stress-associated elements (**Figure 7**; **Supplementary Figure 2**). Several development-related *cis*-elements, such as the CAT-box (associated with meristem development), O2-site (associated with zein metabolism regulation), circadian (associated with circadian control), GCN4_motif (associated with endosperm development), and MSA-like (associated with cell cycle regulation), were located on the promoters of *AsbZIPs*. Ten hormone-responsive regulatory elements associated with ABA, IAA, GA, MeJA, and SA responses, including ABRE (29.48%), TGA-element (8.25%), AuxRR-core (2.59%), TGA-box (0.71%), P-box (5.19%), GARE-motif (3.54%), TATC-box (2.83%), TGACG-motif (19.81%), CGTCA-motif (19.81%) and TCA-element (7.78%), were discovered in the promoters of *AsbZIPs*. Additionally, seven stress-responsive regulatory elements that respond to various stress conditions, such as anaerobic induction, anoxic-specific inducibility, defense and stress responsiveness, drought inducibility, low-temperature responsiveness, salt responsiveness and wound-responsiveness, were identified, including ARE (47.74%), GC-motif (3.01%), TC-rich repeats (8.65%), MBS (20.3%), LTR (19.17%), DRE (0.38%) and WUN-motif (0.75%). These findings indicate that the transcriptional regulation of *AsbZIPs* is associated with development, hormone, and stress.

### Expression patterns of *AsbZIPs* in multiple tissues of garlic

To investigate the function of the *AsbZIP* genes in growth and development, 14 *AsbZIP* genes were randomly selected, and their expression levels in various tissues (bulb, bud, leaf, pseudostem, sprout, root and flower) were determined. As shown in the **Supplementary Figures 3A**, **4** (*Asa6G00995*, *Asa8G02357*, *Asa2G06963* and *Asa4G03115*), and 2 (*Asa8G06181* and *Asa7G01379*) *AsbZIP* genes were relatively highly expressed in roots and leaves, respectively. *Asa6G00995* presented high expression levels in roots, whereas its expression was relatively low in buds. The expression trend of *Asa3G01906* was opposite to that of *Asa6G00995*; *Asa3G01906* presented elevated expression levels in buds, with lower expression levels observed in roots. Additionally, *Asa8G04833* exhibited high expression in flowers, whereas the expression level of *Asa4G00594* was relatively high in bulbs. These distinct expression patterns suggest various roles of these genes in garlic development.

### Expression patterns of *AsbZIPs* under abiotic stress

**Figure 8:**
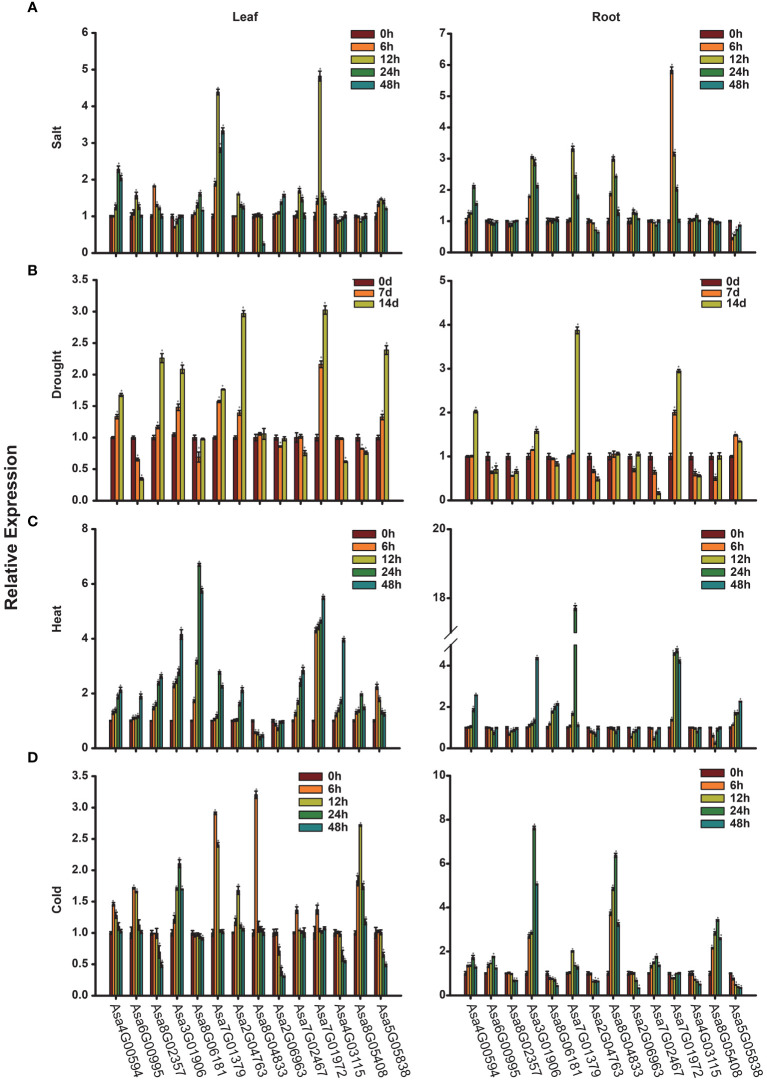
Expression analysis of *AsbZIP* genes. **(A-D)** The expression levels of *AsbZIP* genes under salt **(A)**, drought **(B)**, heat **(C)** and cold **(D)** detected by qRT-PCR. Error bars represent standard deviations from biological replicates. One asterisk (*) denotes a significant difference at *P* < 0.05, determined using Student’s *t*-test.

Given the crucial regulatory roles of bZIP transcription factors in response to adverse conditions, it is imperative to study the expression of *AsbZIPs* under various abiotic stress conditions. **Figures 8A–D** illustrate the expression profiles of *AsbZIP* genes under salt, drought, heat and cold stresses, respectively. Under salt stress, *Asa4G00594*, *Asa7G01379*, *Asa2G06963* and *Asa7G01972* were significantly upregulated in both leaves and roots at different time points. *Asa6G00995*, *Asa8G02357*, *Asa2G04763*, *Asa7G02467* and *Asa5G05838* presented induced expression and peaked at 12 h in leaves but presented suppressed expression in roots under salt stress. In contrast, *Asa3G01906*, *Asa8G04833* and *Asa4G03115* were downregulated in leaves and upregulated in roots at different time points. Additionally, *Asa7G01972* presented the greatest change in gene expression level in both leaves (peaked at 12 h) and roots (peaked at 6 h) under salt stress, followed by *Asa7G01379*, which peaked at 12 h in both leaves and roots. Under drought stress, *Asa4G00594*, *Asa3G01906*, *Asa7G01379*, *Asa7G01972* and *Asa5G05838* were upregulated and peaked at 14 d, whereas *Asa6G00995*, *Asa8G06181*, *Asa2G06963*, *Asa7G02467*, *Asa4G03115* and *Asa8G05408* were downregulated at distinct time points in both roots and leaves, implying their pivotal roles in response to drought stress in distinct tissues. Furthermore, *Asa7G01972* and *Asa7G01379* were the genes whose expression was most predominantly induced in leaves and roots, respectively. Under heat stress, with the exception of *Asa8G04833* and *Asa2G06963*, whose expression decreased, the other genes were induced in the leaves. Moreover, *Asa8G06181* and *Asa7G01379* presented the greatest induction at 24 h in the leaves and roots, respectively. Under cold treatment, *Asa4G00594*, *Asa6G00995*, *Asa3G01906*, *Asa7G01379*, *Asa2G04763*, *Asa8G04833*, *Asa7G02467*, *Asa7G01972* and *Asa8G05408* presented increased expression in the leaves. Among these genes, the expression of *Asa4G00594*, *Asa6G00995*, *Asa7G01379*, *Asa8G04833*, *Asa7G02467* and *Asa7G01972* peaked at 6 h. Seven genes (*Asa4G00594*, *Asa6G00995*, *Asa3G01906*, *Asa7G01379*, *Asa8G04833*, *Asa7G02467* and *Asa8G05408*) were upregulated in the roots at all time points after cold treatment, with *Asa3G01906* exhibiting the highest expression levels. In addition, under cold stress, the majority of *AsbZIPs* were induced in garlic cloves, except for *Asa7G01379*, *Asa8G04833*, and *Asa5G05838* (**Supplementary Figure 3B**). Among these genes, the highest induction level was found in *Asa2G06963* at 40 d, while *Asa5G05838* was the most significantly inhibited gene. Overall, the predominant and distinct expression profiles of these genes suggest their potential roles in the response to abiotic stress.

### Expression analysis of *AsbZIP* genes under hormonal treatment

**Figure 9:**
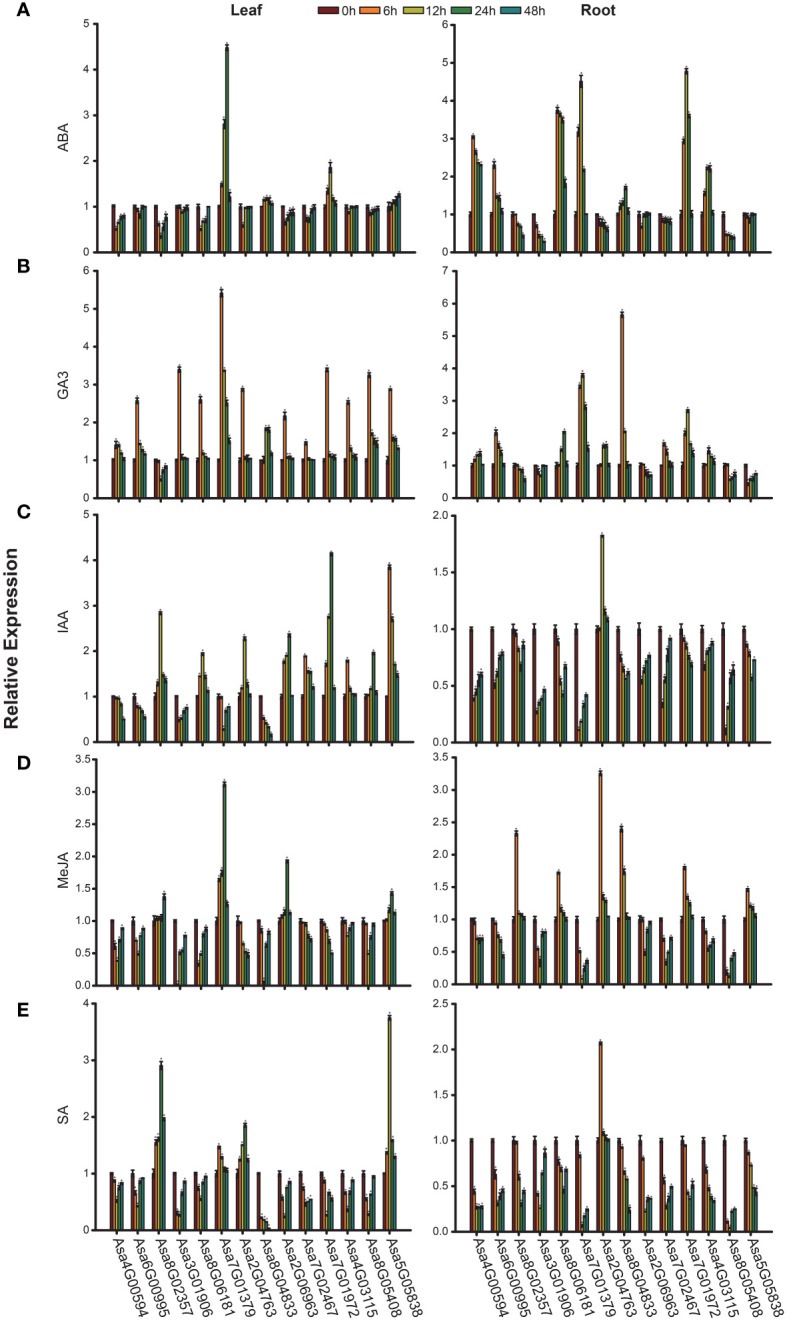
Expression patterns of *AsbZIP* genes under hormone treatment were examined by qRT-PCR. **(A)** Expression profiles of *AsbZIP* genes after exogenous ABA treatment. **(B)** Expression profiles of *AsbZIP* genes after exogenous GA3 treatment. **(C)** Expression profiles of *AsbZIP* genes after exogenous IAA treatment. **(D)** Expression profiles of *AsbZIP* genes after exogenous MeJA treatment. **(E)** Expression profiles of *AsbZIP* genes after exogenous SA treatment. The error bars represent standard deviations from biological replicates. A significance level of *P* < 0.05, as determined by Student’s *t*-test, is indicated by an asterisk (*).

Numerous *cis*-elements associated with hormone response in the promoters of *AsbZIP* genes indicate pivotal roles of these genes in the plant hormone response. Therefore, the expression profiles of *AsbZIP* genes under hormonal stress conditions (ABA, GA3, IAA, MeJA, and SA) were investigated. The expression levels of 14 *AsbZIPs* in garlic leaves and roots at 6 h, 12 h, 24 h and 48 h after treatment were determined via qRT-PCR (**Figures 9A–E**). Under ABA stress, the increased expression patterns of *Asa7G01379*, *Asa8G04833*, *Asa7G01972*, and *Asa5G05838* were detected at all time points, whereas other genes presented reduced expression in leaves. Among these genes, *Asa7G01379* had the highest induction level and peaked at 24 h, followed by *Asa7G01972*, which peaked at 12 h; the most significantly inhibited level was obtained in *Asa8G02357* at 12 h. In roots, *Asa4G00594*, *Asa6G00995*, *Asa8G06181*, *Asa7G01379*, *Asa8G04833*, *Asa7G01972* and *Asa4G03115* were predominantly induced by ABA at all time points, whereas other genes were inhibited. Furthermore, *Asa7G01972* was most significantly induced and peaked at 12 h. Under GA3 treatment, most *AsbZIP* genes presented increased expression profiles, except for *Asa8G02357*, whose expression was most significantly induced in *Asa7G01379* at 6 h in leaves. In roots, the expression levels of 9 *AsbZIP* genes, namely, *Asa4G00594*, *Asa6G00995*, *Asa8G06181*, *Asa7G01379*, *Asa2G04763*, *Asa8G04833*, *Asa7G02467*, *Asa7G01972*, and *Asa4G03115*, were induced by GA3 at different time points. Furthermore, 9 genes (*Asa8G02357*, *Asa8G06181*, *Asa2G04763*, *Asa2G06963*, *Asa7G02467*, *Asa7G01972*, *Asa4G03115*, *Asa8G05408*, and *Asa5G05838*) were upregulated in leaves after IAA treatment. Notably, *Asa7G01972* was strongly induced by IAA treatment, with its expression peaking at 24 h. In roots treated with IAA, only *Asa2G04763* was upregulated, with its expression peaking at 12 h. MeJA stress led to the upregulation of *Asa8G02357*, *Asa7G01379*, *Asa2G06963*, and *Asa5G05838* in leaves, with *Asa8G02357* peaking at 48 h and the other genes peaking at 24 h. After MeJA treatment, the expression of six genes (*Asa8G02357*, *Asa8G06181*, *Asa2G04763*, *Asa8G04833*, *Asa7G01972*, and *Asa5G05838*) in roots increased, and the expression of *Asa2G04763* was notably increased at 6 h. After SA treatment, the expression of *Asa8G02357*, *Asa7G01379*, *Asa2G04763*, and *Asa5G05838* was upregulated in leaves, and only *Asa2G04763* presented increased expression and peaked at 6 h in roots. In summary, these results provide useful information for further functional investigations of *AsbZIPs*.

### Gene regulatory network analysis

Gene regulatory network analysis serves as an effective method for annotating gene function. The regulatory network of *AsbZIP* genes was constructed via WGCNA on the basis of 185 RNA-Seq samples (**Supplementary Table** **7**) and the binding motifs of *bZIP* genes in *Arabidopsis thaliana*, resulting in 31 gene regulatory networks (**Supplementary Table** **8**). The largest network was centered on *Asa7G01972*, which included 201 genes, whereas the smallest network, was centered on *Asa0G04894*, which included 8 genes.

**Figure 10:**
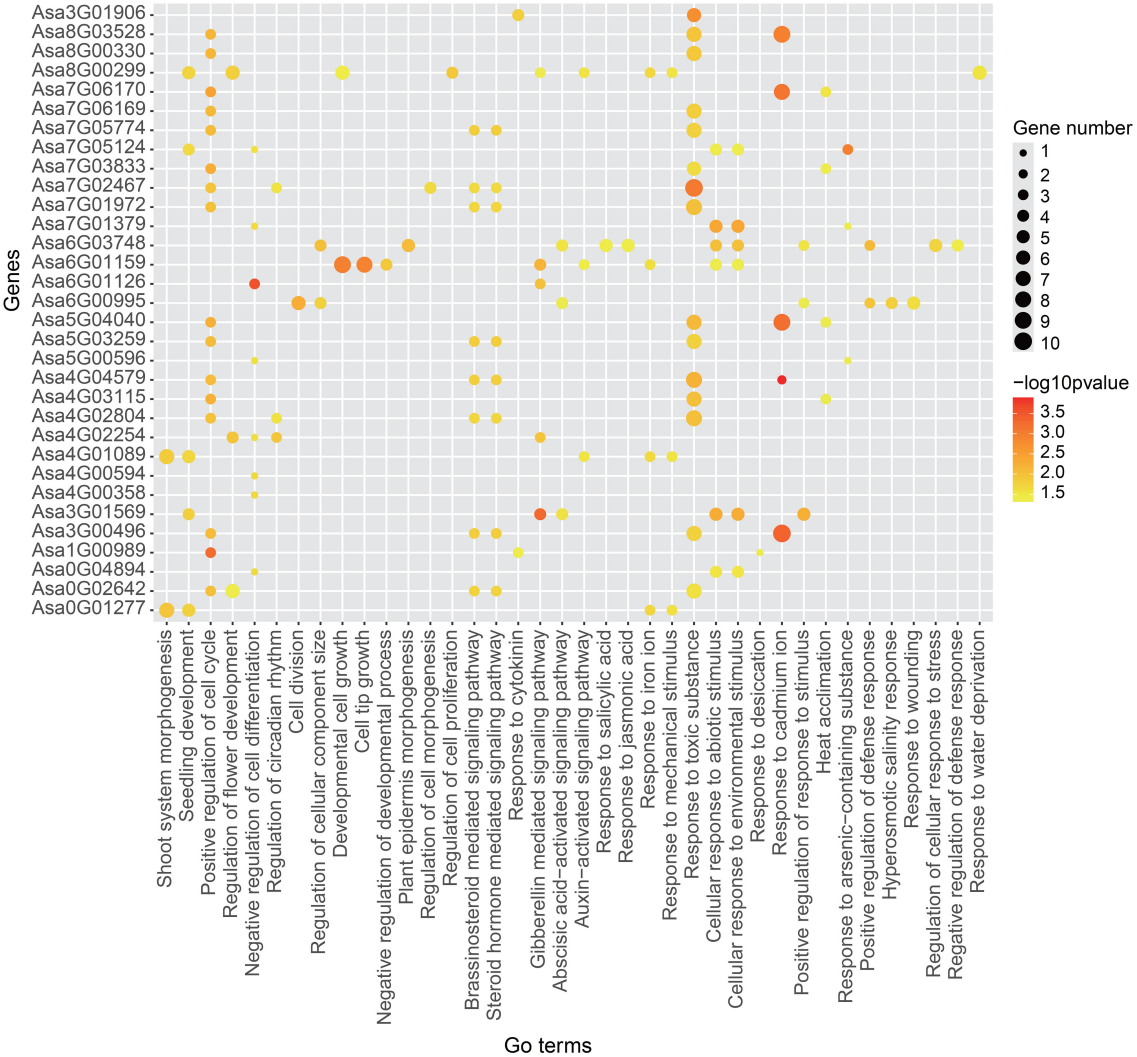
GO enrichment analysis of 31 gene sets of gene regulatory network. The size of each spot represents the number of genes enriched in specific term. The color of each spot indicates the enrichment significance level.

To delve into the potential biological processes in which these genes might be involved, we conducted GO enrichment analysis. GO terms related to stress responses, such as the cellular response to abiotic stimuli, the cellular response to environmental stimuli and heat acclimation, were present in the majority of the *AsbZIP* genes, indicating their vital roles in the stress response (**Figure 10**). In addition to *Asa3G01906*, the gene regulatory networks of *AsbZIP* genes were enriched in the regulation of development and growth, indicating crucial roles of these genes in plant growth and development. Additionally, some GO terms associated with the hormone response, including abscisic acid-activated signaling pathway, gibberellin mediated signaling pathway and response to jasmonic acid, were enriched in the gene regulatory network of several *AsbZIP* genes (*Asa0G02642*, *Asa1G00989*, *Asa3G00496*, *Asa3G01569*, *Asa4G1089*, *Asa4G02254*, *Asa4G02804*, *Asa4G04579*, *Asa5G03259*, *Asa6G00995*, *Asa6G01126*, *Asa6G01159*, *Asa6G03748*, *Asa7G01972*, *Asa7G02467*, *Asa7G05774*, *Asa8G00299* and *Asa3G01906*), indicating that these genes might play critical roles in hormone response regulation.

### AsbZIP protein-protein interaction network prediction

Analyzing the functional associations among AsbZIP proteins is crucial for understanding the regulatory pathways within this protein family. To further elucidate the functions of the AsbZIP proteins, an interaction network of the AsbZIP proteins was constructed (**Supplementary Figure 4**). Asa1G01577 (homologue of ABI5), Asa4G04645 (homologue of ABF3), Asa5G05838 (homologue of ABF4), Asa4G00594 (homologue of ABF2), Asa7G06169 (homologue of ABF1), Asa8G03528 (homologue of DPBF4), Asa7G06185 (homologue of DPBF3) and Asa4G03115 (homologue of DPBF2) are associated with an abscisic acid-activated signaling pathway. Asa6G03748 (GBF4), Asa5G00596 (BZIP68), Asa7G03833 (GBF1), Asa2G05361 (BZIP16) and AT2G46270 (GBF3) are transcription factors regulated by diverse stimuli, such as light-induction or hormone control. Furthermore, Asa7G01379 (HY5) and Asa4G01089 (HYH) are transcription factors that promote photomorphogenesis in light.

### Functional analysis of *Asa7G01379* and *Asa7G01972*

**Figure 11:**
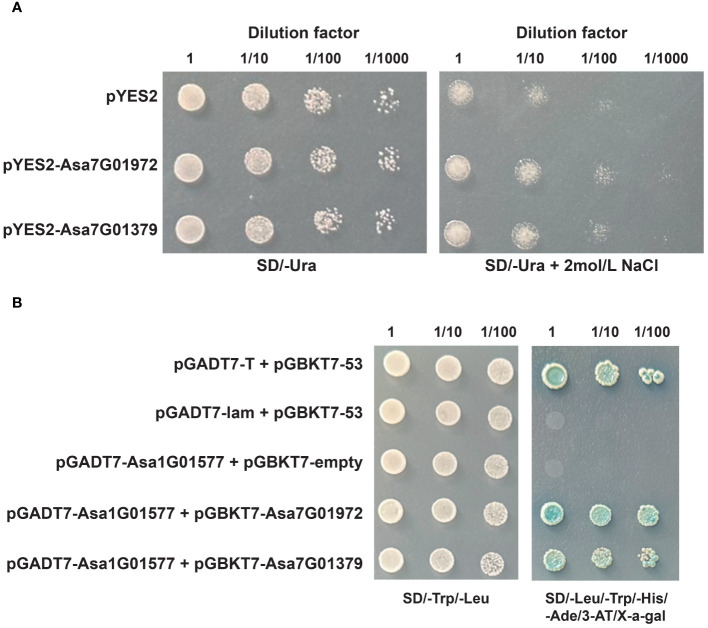
The functional analysis of Asa7G01972 and Asa7G01379. **(A)** The growth conditions of recombinant yeast BY4741 (pYES2-Asa7G01972/pYES2-Asa7G01979) and control yeast BY4741 (pYES2) were assessed under 2 mol/L NaCl treatment for 24 h Photographs of the plates were taken after 72 h of incubation at 30°. **(B)** Yeast two-hybrid analysis. 53-pGBKT7/T-pGADT7 was used as the positive control, and 53-pGBKT7/lam-pGADT7 was used as the negative control.

Owing to their highly significant alterations in expression under salt stress and ABA treatment, *Asa7G01379* and *Asa7G01972* were selected for investigation of their biological functions via heterogeneous expression in yeast (**Figure 11A**). Compared with the yeast carrying the empty pYES2 vector, the growth of the yeast (BY4741) strains containing pYES2-Asa7G01379 and pYES2-Asa7G01972 was not significantly different under the control conditions but was greater under the salt treatment. These results suggest the important roles of *Asa7G01379* and *Asa7G01972* in salt stress.

To confirm the potential protein-protein interaction profiles predicted by STRING, we performed Y2H assays. The results validated the protein interactions of Asa7G01379-Asa1G01577 and Asa7G01972-Asa1G01577 (**Figure 11B**), demonstrating the reliability of the predicted protein interactions. Owing to the vital role of Asa1G01577 (a homologue of ABI5) in the ABA signal transduction pathway, we presume that Asa7G01379 and Asa7G01972 might regulate the response to salt stress through the ABA pathway.

## Discussion

Garlic, with its plump bulbs and high allicin content, is not only used as a vegetable and flavoring ingredient, but is also widely used in the pharmaceutical industry. The *bZIP* gene family is one of the most critical families in plants and plays pivotal roles in regulating plant growth, development, and response to both biotic and abiotic stresses (Rodriguez-Uribe and O’Connell, 2006; Prasad et al., 2012; Gangappa et al., 2013). Previous studies on *bZIP* transcription factors have focused mainly on *Arabidopsis thaliana*, crops and fruits (Jakoby et al., 2002; Nijhawan et al., 2008; Liao et al., 2008a; Liu et al., 2020; Kumar et al., 2021). However, a comprehensive study of *AsbZIP* genes at the genome-wide level in garlic has not been conducted thus far. Here, a comprehensive study of the *bZIP* gene family in garlic was performed, and 64 *bZIP* genes were identified in garlic (genome size: 16 GB). The number of *bZIP* genes in garlic was less than that in *Arabidopsis thaliana* (78, genome size: 117 Mb) (Jakoby et al., 2002), rice (89, genome size: 466 Mb) (Nijhawan et al., 2008), maize (125, genome size: 2182 Mb) (Bolle, 2004), soybean (160, genome size: 915 Mb) (Zhang et al., 2018), and Tartary buckwheat (96, genome size: 489 Mb) (Liu et al., 2019) but greater than that in grape (55, genome size: 490 Mb) (Liu et al., 2014b), indicating that there is no positive correlation between the number of *bZIP* genes and genome size. Phylogenetic analysis revealed that *AsbZIPs* can be divided into 10 subfamilies (**Figure 1**), which was consistent with the findings in *Arabidopsis thaliana* (Jakoby et al., 2002) and grape (Liu et al., 2014b), but lower than those in tartary buckwheat (11) (Liu et al., 2019), Chinese jujube (14) (Zhang et al., 2020b), poplar (12) (Zhao et al., 2021) and potato (11) (Kumar et al., 2021).

Furthermore, gene intron/exon structural analysis revealed variation in the number of introns within *AsbZIP* genes, ranging from 0 to 11 (**Figure 2**), which is similar to that of *bZIP* genes in other plants, such as rice (0-12) (Nijhawan et al., 2008), wheat (0-14) (Liang et al., 2022) and moso bamboo (0-17) (Pan et al., 2019). However, members within the same subfamily shared similar gene structures, supporting the results of the phylogenetic tree. The *bZIP* genes in subfamilies C/E/G have no more than three introns, supporting the hypothesis that genes with fewer introns may facilitate rapid environmental responsiveness (Shabalina et al., 2010). In addition, although there are variations in the motif components of distinct subfamilies, the motifs encoding the bZIP domain are highly conserved, and the majority of members of the same subfamily present similar motif components, which is coincident with the findings of previous studies (Zhou et al., 2017). For example, most AsbZIP proteins in subfamily S share motifs 1 and 6, with the exception of Asa5G04040, which only possesses motif 1. Several motifs (such as motifs 8, 9, and 10) found exclusively in specific subfamilies might contribute to the diverse biological functions of *AsbZIP* genes, which is consistent with previous reports (Wang et al., 2018; Liu et al., 2019).

The expansion of gene families through tandem or segmental duplication is crucial for functional diversity in evolution (Cannon et al., 2004). Furthermore, another study indicated that garlic has experienced three whole genome duplication events (Sun et al., 2020). Here, two tandem duplications and seven segmental duplications of *AsbZIPs* were identified in the garlic genome (**Figures 3**, **4**), suggesting the vital role of gene replication in *AsbZIP* gene family expansion, which is consistent with previous reports (Nijhawan et al., 2008; Liu et al., 2014b, 2019). The number of paralogous pairs in garlic (9) was less than that in poplar (31) (Zhao et al., 2021), rice (34) (Nijhawan et al., 2008) and pear (68) (Ma et al., 2021). Additionally, the results of evolutionary analysis between garlic *bZIP* genes and their counterparts in two eudicots and two monocots revealed that there were more collinear gene pairs between garlic and *Oryza sativa* (**Figure 5**), indicating relatively close evolutionary relationships between these species.

The exposure of wild species to novel selection environments driven by human demands gradually induces different morphological and physiological alterations, eventually leading to their divergence from wild ancestors. This process represents domestication, a form of coevolution between plants and animals (Purugganan and Fuller, 2009). Cultivated garlic, which originates from the wild *Allium longicuspis* and *Allium tuncelianum*, has undergone genetic alterations leading to divergence in plant architecture and growth habits, referred to as the domestication syndrome (Li et al., 2022). Nevertheless, the alterations in *AsbZIPs* resulting from garlic domestication are poorly understood. Here, we identified 2941 *AsbZIP*-associated SNPs and observed their uneven distribution across the genome sequence (**Supplementary Table** **6**), including a total of 2783 intergenic and 158 intragenic variations, which was consistent with the report presenting lower polymorphism of SNPs in intragenic regions than in intergenic regions (Hao and He, 2024). PCA, admixture and phylogenetic analysis successfully segregated all the accessions into two groups: wild garlic and local garlic (**Figures 6A, B**), which was consistent with the phylogenetic classification between wild and domesticated garlic of *AsHSFs* (Hao and He, 2024). In addition, domestication of garlic led to a bottleneck that decreased nucleotide diversity in alleles. The nucleotide diversity of *AsbZIPs* in wild garlic was slightly higher than that of domesticated garlic, with a decrease of only 0.00003%, which is lower than the average reduction of nucleotide diversity from wild garlic to landraces (Li et al., 2022), indicating a minor genetic bottleneck in *AsbZIP* genes during domestication. This result was confirmed by the Fst result, implying that *AsbZIP* genes did not experience strong selection pressure during domestication.

*bZIP* genes are related to plant development, and analyzing tissue-specific gene expression profiles facilitates to further comprehension of the biological functions of these genes. In *Arabidopsis thaliana*, HY5 (AtbZIP56) interacts with BBX25 to inhibit seedling photomorphogenesis through regulating the gene expression of *BBX22* (Gangappa et al., 2013). In *Oryza sativa*, overexpression of *OsbZIP49* resulted in the reduction of internode length and plant height, showcasing a tiller-spreading phenotype (Gangappa and Botto, 2016; Ma et al., 2018; Lorenzo, 2019; Ding et al., 2021; Han et al., 2023; Liu et al., 2023). Moreover, suppressed plant growth and decreased number of petals were observed in the *Arabidopsis thaliana* overexpressing the *Capsicum annuum CabZIP1* gene (Lee et al., 2006). Here, we systematically investigated the expression profiles of *AsbZIPs* in several tissues of garlic. As shown in **Supplementary Figure 3A**, *Asa2G04763*, *Asa7G02467*, *Asa8G05408* and *Asa3G01906* were highly expressed in floral buds, and *Asa6G00995*, *Asa8G02357*, *Asa4G03115* and *Asa2G06963* presented relatively high expression levels in roots, whereas relatively high expression levels in leaves were observed for *Asa8G06181* and *Asa7G01379*, suggesting potential specific functions of these genes in particular organs during the growth and development of garlic, which was comparable with tissue-specific expression profiles of *bZIP* genes in other species including *Malus halliana* (Wang et al., 2021b), *Musa nana* (Hu et al., 2016a) and *Citrullus lanatus* (Yang et al., 2019).

In addition, numerous studies have revealed that *bZIP* TFs are involved in responding to stresses. In wheat, *TabZIP96* played a crucial role in the regulation of cold stress (Liang et al., 2022). In potato, the gene expression of *StbZIP25* was induced under salt stress and could improve salt tolerance (Wang et al., 2021a). In pepper, *CabZIP25*-overexpression resulted in increased fresh weight and root length under salt stress (Gai et al., 2020). In *Oryza sativa*, *OsbZIP71* could regulate the expression of *OsNHX1* and *COR413-TM1* to improve the drought and salinity tolerance (Liu et al., 2014a). In *Vitis vinifera*, the expression of *VvbZIP23* was upregulated by several abiotic stresses, such as cold and drought stresses (Tak and Mhatre, 2013). Our results revealed that most *AsbZIP* genes were specifically induced or repressed under multiple types of stress (**Figures 8A, D**). In particular, *Asa4G00594* is upregulated under salt and drought stresses, and its homologous gene *AtABF2* is known to contribute to the tolerance to salinity and drought stresses (Fujita et al., 2005; Du et al., 2023). Moreover, *Asa7G01379*, encoding the AsHY5 protein, was upregulated significantly under salt stress, which is coincident with the findings of a previous report (Yang et al., 2023). The most of *AsbZIP* genes were induced under heat stress, except for *Asa8G04833* and *Asa2G06963*. Under cold stress, the expression levels of *Asa7G01379* and *Asa2G06963* increased and decreased, respectively, which is consistent with the key roles of their homologues in cold acclimation in *Arabidopsis thaliana* (Oono et al., 2006; Perea-Resa et al., 2017). The expression profiles of *AsbZIP* genes under abiotic stresses provide potential avenues for garlic breeding to increase their ability to tolerance stresses.

An increasing number of studies have revealed that *bZIP* genes are involved in phytohormone signaling. In rice, *OsbZIP72* and *OsbZIP23* were identified as regulators of ABA response to affect drought tolerance (Xiang et al., 2008; Lu et al., 2009). In *Arabidopsis thaliana*, *AtbZIP39*, *AtbZIP36*, *AtbZIP38*, *AtbZIP35* and *AtbZIP37* have been found to play vital roles in the ABA signaling pathway (Choi et al., 2000; Uno et al., 2000; Lopez-Molina et al., 2001; Hossain et al., 2010; Liang et al., 2022). In *Artemisia annua*, the *bZIP* gene *AaTGA6* was demonstrated as a key regulator in the SA signaling pathway (Lv et al., 2019), and *AabZIP1* played a vital role in the regulation of ABA signaling (Zhang et al., 2022a). Here, the identification of *cis*-acting elements revealed the potential roles of *AsbZIPs* in phytohormone signal transduction (**Figures 7A, B**; **Supplementary Figure 2**). As shown in **Figures 9A-E**, the majority of *AsbZIP* genes showed significant alteration under several hormone stresses. For example, after ABA treatment, *Asa4G00594* and *Asa5G05838* were induced in roots and leaves, respectively, which is consistent with previous investigations (Choi et al., 2000; Kim et al., 2004). Particularly, *Asa7G01379* and *Asa7G01972* were the most significantly induced *AsbZIP* genes under salt stress, implying crucial functions of these genes for salt stress response. Furthermore, GO enrichment terms of gene regulatory network analysis showed that *Asa7G01379* and *Asa7G01972* might be involved in the stress response (**Figure 10**), which was confirmed by yeast-induced expression assay (**Figure 11A**). Besides, the Y2H experiment demonstrated the interactions of Asa7G01379-Asa1G01577 and Asa7G01972-Asa1G01577, as predicted by STRING (**Figures 11B**; **Supplementary Figure 4**), which is useful for revealing the biological functions of these AsbZIP proteins involved in the stress response through ABA signaling. According to the above results, *Asa7G01379* and *Asa7G01972* might be potential target locus for breeding stress-resistant garlic.

## Conclusions

Here, we systematically investigated the genome-wide *AsbZIP* TFs in garlic. We identified 64 *AsbZIP* genes and conducted a comprehensive analysis of their physical characteristics, evolutionary associations, gene structures, conserved motifs, gene duplication events, nucleotide variation, population structure, expression patterns, and responses to abiotic stress and hormone treatment. On the basis of the above analysis, we speculate that *AsbZIP* genes play vital roles in the development and response of garlic to stress. Furthermore, our study revealed that 2 ABA-responsive genes, *Asa7G01972* and *Asa7G01379*, are closely related to the response to salt stress in garlic. This study could inform subsequent functional analysis of *AsbZIP* genes, contributing to a more in-depth understanding of the molecular mechanisms underlying developmental processes and stress responses in garlic.

## Data availability statement

The original contributions presented in the study are included in the article/**Supplementary Material**. Further inquiries can be directed to the corresponding author.

## Author contributions

SH: Conceptualization, Data curation, Funding acquisition, Investigation, Writing – original draft, Writing – review & editing. SX: Data curation, Investigation, Writing – review & editing. ZH: Data curation, Investigation, Writing – review & editing. XH: Conceptualization, Data curation, Funding acquisition, Investigation, Writing – review & editing.
