# Supplementary material for: Genome-wide identification, characterization and expression analysis of the bZIP transcription factors in garlic (Allium sativum L.)
Source: Front Plant Sci. 2024 Aug 1;15:1391248. doi: 10.3389/fpls.2024.1391248 (PMC11324451; doi:10.3389/fpls.2024.1391248)
Supplement: Supplementary file 1 [file DataSheet_1.docx]

**Supplementary Fig. 1.**

**
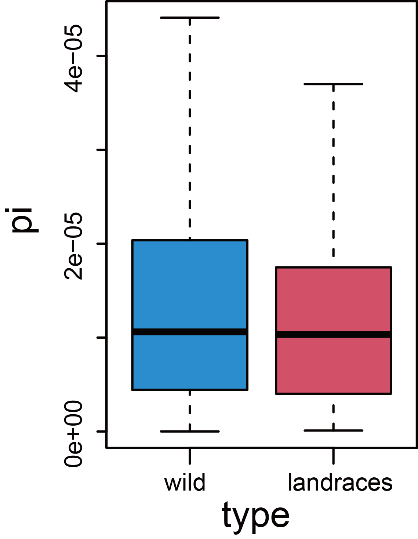
**

**Supplementary Fig. 1.** Nucleotide diversity in wild garlic and landrace accessions. The bottom, middle, and top lines in the box denote the first, median, and third quartiles of the distribution, respectively. Nucleotide diversity was calculated based on *AsbZIP*-related SNPs.

**Supplementary Fig. 2.**

**
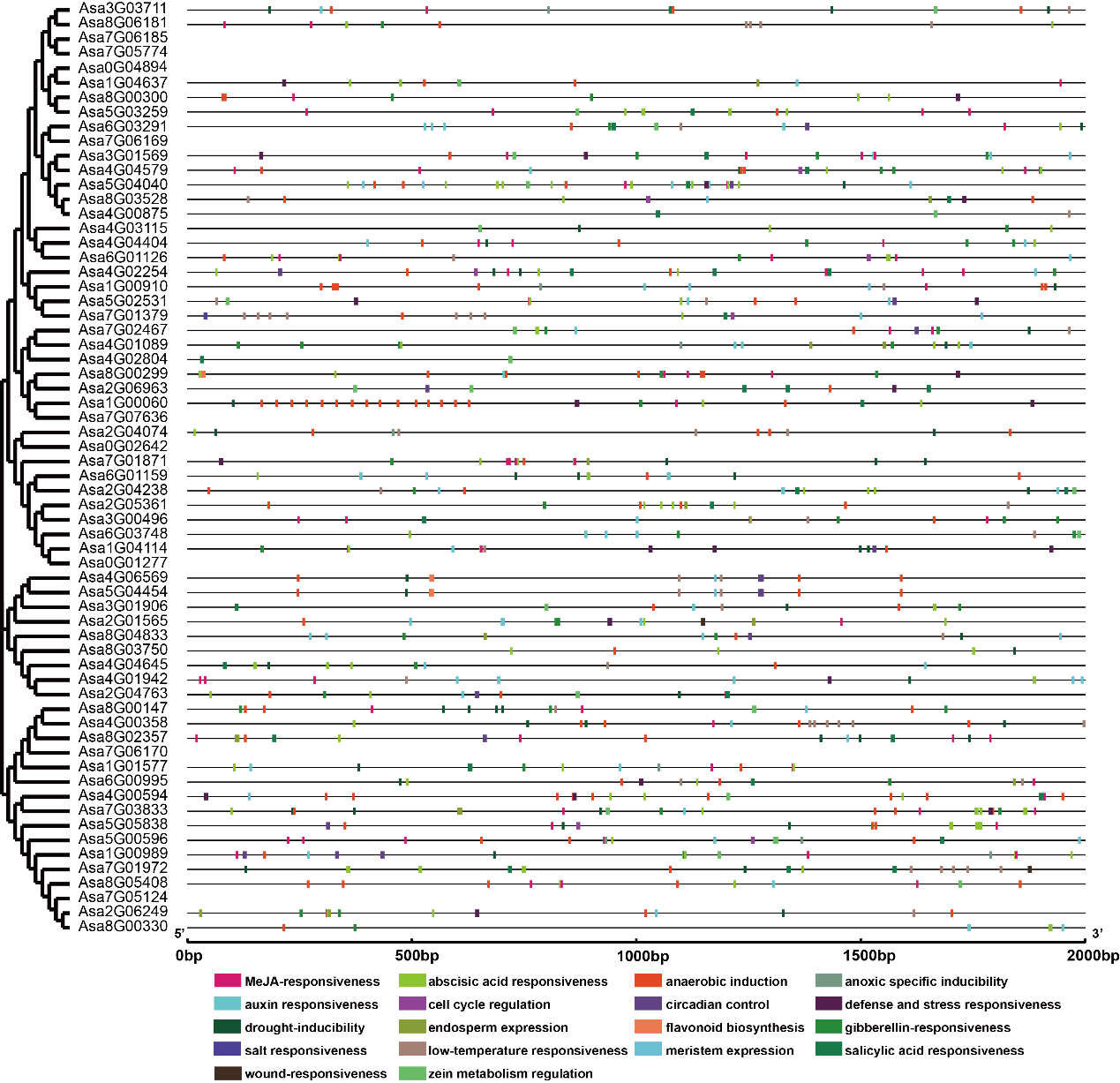
**

**Supplementary Fig. 2.** Phylogenetic relationships and *cis*-elements in the promoters of the *AsbZIP* genes. Each *cis*-acting element is depicted by a rectangular box of a specific color.

**Supplementary Fig. 3.**

**
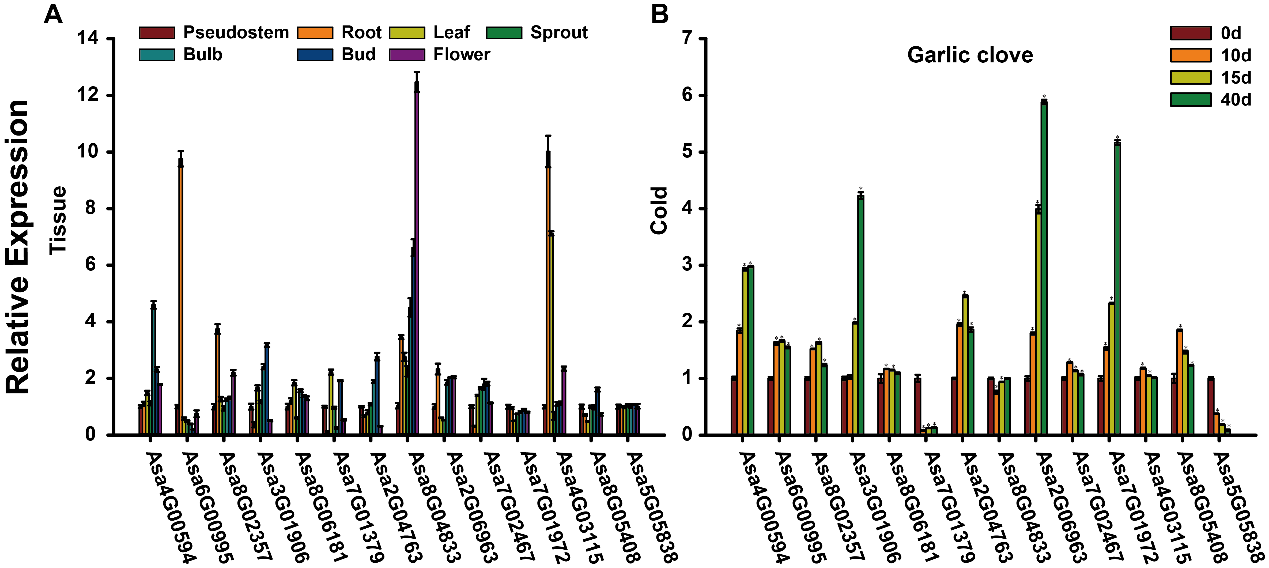
**

**Supplementary Fig. 3.** Expression analysis of *AsbZIP* genes. (a) The expression patterns of *AsbZIP* genes in various tissues analysed by qRT-PCR. (b) The expression levels of *AsbZIP* genes in garlic clove under cold detected by qRT-PCR. Error bars represent standard deviations from biological replicates. One asterisk (*) denotes a significant difference at *P* < 0.05, determined using Student’s *t*-test.

**Supplementary Fig. 4.**

**
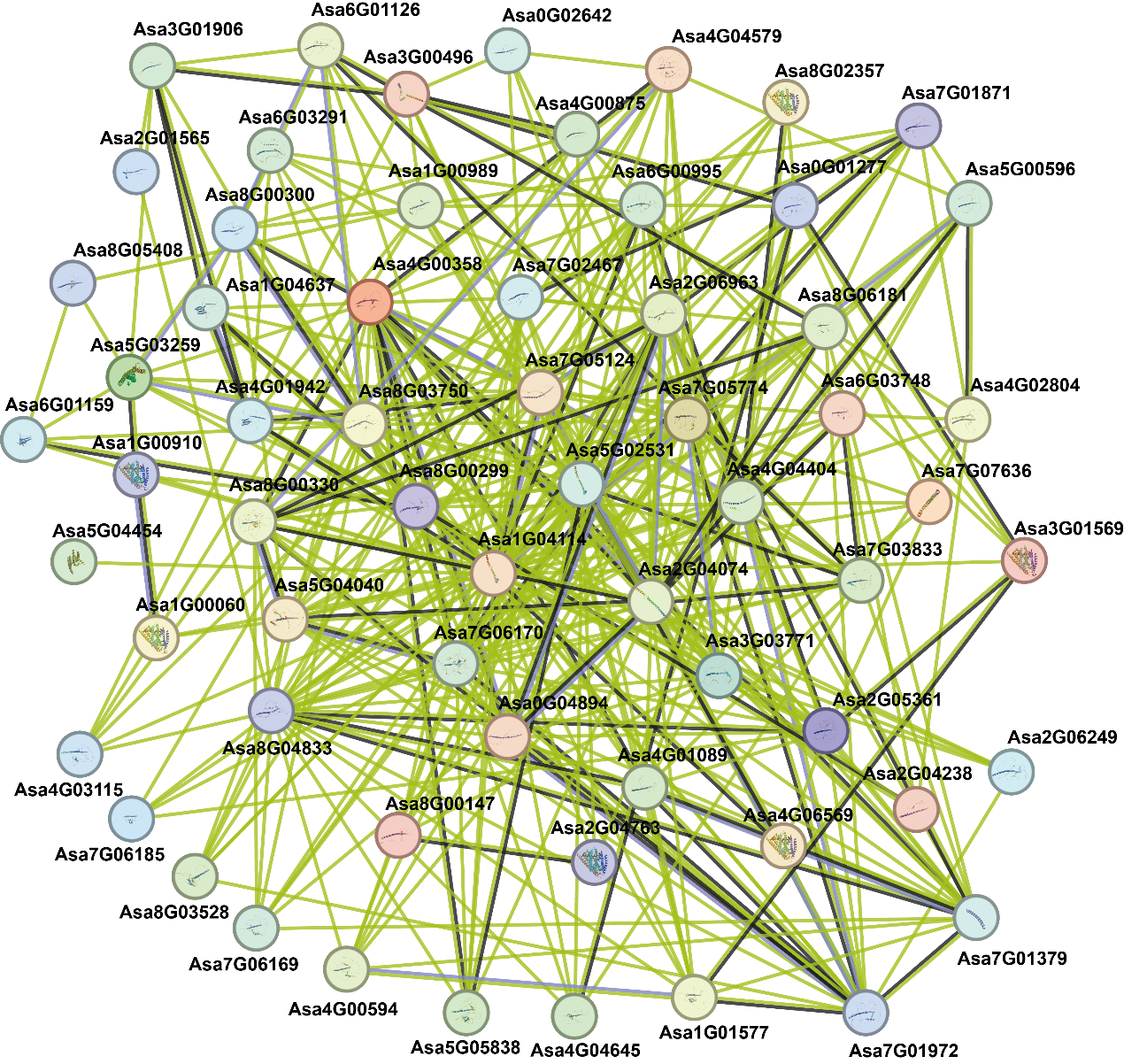
**

**Supplementary Fig. 4.** Protein-protein interaction network of AsbZIPs based on their orthologs in *Arabidopsis thaliana*. Network nodes symbolize proteins, with the 3D protein structure displayed within the nodes, while lines denote protein-protein interactions.
